# The investigation and analysis of nutritional iron deficiency anaemia in Kazakh children

**DOI:** 10.3389/fped.2025.1654351

**Published:** 2026-01-12

**Authors:** Yu Wei, Jianghong Wang, Feng Wang, Xu Wang, Xiufang Cheng

**Affiliations:** 1 Department of Pediatrics, The People’s Hospital of Anyang City, Anyang, Henan, China; 2 Department of Internal Medicine, Barkol Kazakh Autonomous County People’s Hospital, Hami, Xinjiang, China

**Keywords:** iron deficiency anaemia, children, risk factor, Kazakh, dietary iron (Fe)

## Abstract

**Objective:**

This study aimed to investigate nutritional iron deficiency anaemia (IDA) in Kazakh children.

**Method:**

In this cross-sectional study, a total of 197 children aged 3–14 years were randomly selected from three townships in Barkol County, and the basic information and diet of the sample children were collected and analysed.

**Results:**

The overall prevalence of IDA was 15.7%, which is considerably higher than the national average for children in China. Kazakh children had a significantly higher prevalence of anaemia than their Han counterparts (*p* = 0.040). To ensure statistical stability given the sample size, a parsimonious multivariate logistic regression model was employed. This model identified lower intake of meat [adjusted odds ratio (aOR) = 0.92], lower intake of aquatic products (aOR = 0.35), and failure to take iron supplements (aOR = 5.10) as independent risk factors for IDA.

**Conclusions:**

The findings indicate a substantial burden of IDA among Kazakh children in this region, highlighting a significant ethnic disparity. The primary modifiable risk factors are dietary, centring on insufficient consumption of bioavailable iron from animal sources and aquatic products, coupled with a lack of iron supplementation. The counterintuitive association between soybean/nut intake and IDA risk warrants further investigation. These results underscore the urgent need for targeted nutritional interventions and health education programmes focused on improving dietary iron quality and supplementation practices in this population.

## 1 Introduction

Iron deficiency anaemia (IDA) is a type of anaemia characterised by reduced haemoglobin (Hb) synthesis due to insufficient iron reserves in the body (1). Iron is an essential trace element for almost all species, including most bacteria, and is also an essential cofactor for many enzymes of energy and oxidative metabolism (2). It participates in numerous critical metabolic processes, including oxygen transport, mitochondrial electron transport, DNA replication and repair, cell signal transduction and free radical generation (3). The aetiology of iron deficiency in children is multifactorial, encompassing causes such as maternal iron deficiency, increased iron demands during rapid growth phases, insufficient dietary intake of iron-rich foods, impaired iron absorption and chronic blood loss leading to excessive iron depletion (4).

According to the World Health Organization (WHO), approximately 2 billion people worldwide suffer from anaemia, of whom 43% are children aged 0–5 years, and about half are caused by iron deficiency. At the beginning of the 21st century, the prevalence of IDA in children aged <7 years in China was 7.8%, and the prevalence of IDA in infants was as high as 20.5%. Although this proportion has declined compared with the 1990s, it is still higher than that of developed countries (3). A meta-analysis showed (5) that the total prevalence of children aged 0–14 years in China was 19.9%, with the highest prevalence in infancy (30.3%). The prevalence rate of rural children (25.6%) is much higher than that of urban children (9.1%), especially in the western rural areas. The incidence of anaemia among children aged 0–6 years in Xinjiang in 2018 was 25.09%, which was 1.88% higher than 23.21% in 2017 and 13.49% higher than the national average of 11.6% (6). At the same time, a previous study showed that in some areas of Xinjiang, anaemia in children is very serious, with 82.79% of the cases being IDA (7). Although previous studies have indicated a high burden of anaemia in Xinjiang, particularly in rural and ethnic minority areas (6, 7), there is a lack of direct comparisons within these regions. To better understand the specific factors associated with IDA in Kazakh children, this study includes a comparison group of Han children from the same county. This internal comparison helps to isolate ethnicity-specific dietary and lifestyle factors from broader regional socioeconomic or environmental influences. The current study shows that the current situation of IDA in children in Xinjiang is still very serious, and effective measures should be taken to prevent and treat it.

The diagnosis and treatment of IDA is not a difficult task in clinical work; however, if we only meet the symptomatic treatment—that is, ‘cure’—and ignore the treatment of the cause, not only is the best treatment time missed due to the delay of the change of the disease but also this leads to the repeated recurrence of anaemia. Therefore, early detection of the cause of IDA and timely treatment and control is an important part of the diagnosis and treatment of IDA.

The Kazakh children in the agricultural and pastoral areas of Barkol County are a population of particular concern. Their specific living environment and traditional pastoral lifestyle shape distinctive dietary patterns, which may differ significantly from national recommendations and contribute to unique IDA risk factors (8). However, existing studies on childhood IDA in Xinjiang have primarily described the overall high prevalence or focused on single ethnic groups (6, 7), leaving a critical gap: a lack of comparative studies between ethnic groups within the same region. Such a comparison is essential to isolate ethnicity-specific dietary and lifestyle factors from broader regional socioeconomic or environmental influences. Therefore, this study is designed to investigate and compare the incidence and related factors of IDA between Kazakh and Han children in Barkol County. The findings aim to fill this knowledge gap and provide an evidence base for formulating targeted and effective prevention strategies.

## 2 Materials and method

### 2.1 Study participants

This cross-sectional study was conducted between June 2024 and June 2025. A total of 200 children aged 3–14 years were selected using a stratified random sampling method from three townships in Barkol County. The sampling strategy aimed to enable an internal comparison by including both Kazakh children, who are the primary ethnic group in this autonomous county, and Han Chinese children residing in the same areas. This allowed for an investigation into whether IDA prevalence and associated factors differed by ethnicity within the same geographic and healthcare setting. The final sample consisted of 150 Kazakh and 47 Han children. The primary outcome of this study was the presence or absence of nutritional iron deficiency anemia (IDA). The diagnostic criteria for IDA in children, as formulated by the editorial committee of the Chinese Journal of Pediatrics and other expert groups, were as follows (4): (1) clear reasons for iron deficiency, such as insufficient iron intake, difficulty in absorption, increased body demand for iron or chronic blood loss; (2) Hb < 110 g/L (anaemia severity classification—light: 90 ≤ Hb < 110 g/L; moderate: 60 ≤ Hb < 90 g/L; severe: Hb < 60 g/L); (3) peripheral blood red blood cells showed small cell hypochromic changes—mean corpuscular volume (MCV) < 80 fL, mean corpuscular participant (MCH) < 27 pg, mean corpuscular participant concentration (MCHC) < 310 g/L; (4) the following four indicators of iron metabolism at least two abnormalities: (i) serum ferritin (SF) < 15 μg/L; (ii) serum iron < 10.7 μmol/L (60 μg/dL); (iii) total iron-binding capacity > 62.7 μmol/L (350 μg/dL); (iv) transferrin saturation < 15%. Those who meet the above diagnostic criteria in items (2) and (3), namely the presence of small cell hypochromic anaemia, combined with a clear cause of iron deficiency, can be diagnosed as IDA. If the iron metabolism index also meets item (4), it can be diagnosed as IDA. At the same time, according to the health industry standards and WHO recommendations on the participant concentration of anaemia, the anaemia rate was calculated according to the altitude adjustment (based on the average altitude of the township where the survey was conducted). The altitude-adjusted anaemia diagnostic criteria = original diagnostic criteria×[1 + 4% × altitude (m)/1,000] (9).

The exclusion criteria were as follows: (1) children with anaemia caused by non-nutritive factors, such as congenital diseases, endocrine system diseases and organ functional diseases, or children with other genetic metabolic diseases or acute and chronic inflammatory diseases; (2) intake of drugs that may affect iron levels within 1 month before the survey; (3) unqualified or missing blood samples; (4) patients with incomplete or missing questionnaires. Potential confounders considered in this study included demographic factors (e.g., age, gender, ethnicity), dietary intake of various food groups (e.g., cereals, meats, dairy, vegetables, fruits), and iron supplementation status.

This study was approved by the hospital ethics committee. Written informed consent was obtained from all parents/local guardians.

### 2.2 Study method

In this study, a self-made questionnaire was used to collect the basic information and diet of the sample children in a face-to-face manner, combined with the local eating habits and characteristics. At the same time, the 24-hour dietary review method was used to investigate the food intake of the sample children for 3 consecutive days. During the survey, food maps and food models were used to help the caregivers of the sample children and the head of the kindergarten canteen to review and estimate the children's food intake in order to obtain as complete and accurate dietary information as possible.

Venous blood was taken from all children to allow blood cell analysis (white blood cell count, Hb, MCV, MCH, MCHC) and serum albumin and serum prealbumin detection; children in the anaemia group completed SF determination. Blood cell analysis was performed using a BC-5390CRP automatic blood cell analyser of Shenzhen Mindray Biotechnology Co., Ltd., Shenzhen, China; serum albumin and prealbumin were measured using an AU5800 automatic biochemical analyser of Beckman Coulter, Brea, California, USA; high-sensitivity C-reactive protein was measured using a PA-900 (Aristo) specific protein analyser of Beijing Zhonghai Shengtai Company, Beijing, China; serum ferritin was measured using a MAGLUMIX8 automatic chemical immunoassay analyser of Shenzhen New Industry Biological Company, Shenzhen, China.

### 2.3 Quality control

Standardised training: organised the systematic study of knowledge related to IDA, systematically explained the principles, methods, precautions and specific operation procedures of the survey to the staff; required the trained personnel to conduct on-site operation demonstrations, corrected the improper operation and finally carried out theoretical and operational assessments for all trained personnel. Only qualified personnel could participate in the survey. Regularly carried out theoretical and operational assessments for the staff participating in the survey, and gave timely training to those who failed to pass the assessment and suspended the qualification of the survey. There was no difference between the survey content and the specific operation.

### 2.4 Statistical analysis

Statistical analysis was performed using SPSS 26.0 statistical software. The normality test was performed using the Kolmogorov–Smirnov method. The measurement data that satisfied the normality were expressed as mean ± standard deviation (x ± s). The independent sample *t-*test was used for group design. The mean comparison between multiple groups was performed using one-way analysis of variance, and the least significant difference method was used for pairwise comparison. The count data were expressed as frequency (*n*) or rate (%). The *χ*^2^ test was used for those who met the conditions, and the Fisher exact probability method was used for those who did not meet the conditions. To control for potential confounding effects and mitigate overfitting due to the limited number of anaemia cases (*n* = 31), rigorous steps were taken in the regression analyses. First, univariate logistic regression was performed to identify variables associated with IDA for preliminary screening (significance level set at *p* < 0.1). Subsequently, to provide robust and reliable estimates, a multivariate logistic regression model was constructed by including only a limited number of the most clinically relevant and statistically significant variables. The construction of this model strictly adhered to the rule of thumb of events per variable (EPV) > 10, which limited the final model to a maximum of three predictors. Interaction effects between key variables (e.g., ethnicity and dietary factors) were tested by including product terms in the models. The goodness-of-fit of the logistic model was assessed using the Hosmer–Lemeshow test. A two-sided *p*-value of <0.05 was considered statistically significant for all final analyses. The handling of missing data followed a complete case analysis approach. Cases with unqualified/missing blood samples or incomplete/missing questionnaires were excluded from the final analysis.

## 3 Results

### 3.1 General data

A total of 197 valid participants were included in this study. One child's blood sample was unqualified, and two children were excluded due to strong rejection of blood sampling. There were 109 boys and 88 girls, 31 cases of anaemia and 166 cases of non-anaemia, with an average age of 7.34 ± 3.30 years. The flow of participants through the study is detailed in Supplementary Figure S1.

### 3.2 Comparison of anaemia in different nationalities

The study included 150 Kazakh children and 47 Han children. Kazakh children had a significantly lower mean corpuscular participant concentration (334.11 ± 16.89 vs. 340.11 ± 12.08, *p* = 0.025) and a higher red blood cell distribution width (14.14 ± 1.89 vs. 13.06 ± 0.70, *p* < 0.001) than Han children. Critically, the prevalence of anaemia was significantly higher among Kazakh children (29/150, 19.3%) than among Han children (2/47, 4.3%) (*p* = 0.040). For detailed haematological parameters, see Table 1.

**Table 1:** Comparison of anemia status by ethnicity.

Item	Kazakh(*n* = 150)	Han(*n* = 47)	*P* value
Erythrocyte total	4.82 ± 0.42	4.71 ± 0.34	0.110
Hemoglobin content	130.85 ± 26.93	133.53 ± 9.73	0.505
Hemotocrit	38.50 ± 3.82	39.29 ± 2.95	0.195
Mean corpuscular volume	80.09 ± 7.45	83.60 ± 3.06	0.002
Mean corpuscular hemoglobin concentration	30.14 ± 28.89	28.41 ± 1.27	0.682
Mean corpuscular hemoglobin concentration	334.11 ± 16.89	340.11 ± 12.08	0.025
Red blood cell distribution width	14.14 ± 1.89	13.06 ± 0.70	<0.001
Anemia status			0.040*
Without	121	45	
Light	24	2	
Moderate to severe	5	0	

* Fisher's exact probability method was used.

### 3.3 Comparison of anaemia status in different genders

The results showed that there were 109 boys and 88 girls. No statistically significant differences were observed in the total number of red blood cells, participant content, haematocrit or mean red blood cell volume between boys and girls (all *p* > 0.05), as shown in Table 2.

**Table 2:** Comparison of anemia status in different genders.

Item	Male(*n* = 109)	Female(*n* = 88)	*P* value
Erythrocyte total	4.80 ± 0.41	4.78 ± 0.39	0.760
Hemoglobin content	130.04 ± 12.41	133.30 ± 33.13	0.344
Hemotocrit	38.49 ± 3.29	38.93 ± 4.04	0.398
Mean corpuscular volume	80.43 ± 6.76	81.54 ± 6.92	0.261
Mean corpuscular hemoglobin concentration	27.21 ± 2.69	32.85 ± 37.48	0.119
Mean corpuscular hemoglobin concentration	337.45 ± 14.39	333.18 ± 17.70	0.063
Red blood cell distribution width	13.84 ± 1.67	13.94 ± 1.84	0.681
Anemia status			0.666*
Without	91	75	
Light	16	10	
Moderate to severe	2	3	

* Fisher's exact probability method was used.

### 3.4 Comparison of anaemia status in different age groups

The study participants were categorized into three age groups: 68 children aged 2–5 years (including 16 anaemia cases, comprising 12 light and 4 moderate to severe cases), 111 children aged 6–12 years (including 14 anaemia cases, comprising 13 light and 1 moderate to severe case), and 18 children aged 13–14 years (including 1 light anaemia case). There were statistically significant differences in haematocrit, mean corpuscular volume and red blood cell distribution width among the three groups, as shown in Table 3.

**Table 3:** Comparison of anemia status in different age groups.

Item	2–5 year(*n* = 68)	6–12 year(*n* = 111)	13–14 year(*n* = 18)	*P* value
Erythrocyte total	4.84 ± 0.47	4.77 ± 0.34	4.80 ± 0.45	0.521
Hemoglobin content	128.49 ± 37.91	132.15 ± 10.83	138.78 ± 10.30	0.246
Hemotocrit	37.23 ± 3.70	39.29 ± 3.35	40.46 ± 3.46	<0.001
Mean corpuscular volume	77.38 ± 8.90	82.54 ± 4.58	84.36 ± 3.26	<0.001
Mean corpuscular hemoglobin concentration	29.66 ± 31.38	29.95 ± 23.06	28.58 ± 1.63	0.977
Mean corpuscular hemoglobin concentration	333.10 ± 15.38	336.53 ± 16.82	338.67 ± 13.00	0.264
Red blood cell distribution width	14.62 ± 2.45	13.48 ± 1.08	13.61 ± 0.86	<0.001
Anemia status				0.112*
Without	52	97	17	
Light	12	13	1	
Moderate to severe	4	1	0	

* Fisher's exact probability method was used.

### 3.5 Interaction analysis

The results showed that there was no interaction between ethnic * age group, ethnic * gender, ethnic * age group * gender, age group * gender (*p* > 0.05), as shown in Table 4.

**Table 4:** Interaction analysis.

Item	Mean square	*F* value	*P* value
Ethnicity × age group	0.135	1.051	0.352
Ethnicity × gender	0.045	0.351	0.554
Ethnicity × age group × gender	0.008	0.060	0.806
Age group × gender	0.066	0.511	0.601

### 3.6 Anaemia and non-anaemia food intake and iron status

The results showed that the intakes of cereals (150.58 ± 6.59 vs. 156.82 ± 8.29, *p* < 0.001), milk and dairy products (493.94 ± 12.28 vs. 501.10 ± 15.56, *p* < 0.001), vegetables (104.13 ± 16.82 vs. 108.57 ± 9.09, *p* = 0.034), fruits (126.74 ± 15.58 vs. 133.34 ± 11.37, *p* = 0.006), eggs (50.61 ± 5.68 vs. 54.61 ± 7.17, *p* = 0.004), meat (53.71 ± 14.22 vs. 60.33 ± 12.55, *p* = 0.009) and aquatic products (1.03 ± 1.02 vs. 2.64 ± 1.26, *p* < 0.001) in the anaemia group were lower than those in the non-anaemia group. The intake of soybean and nuts (22.74 ± 4.97 vs. 18.46 ± 5.35, *p* < 0.001) were higher than those without anaemia; among them, the intakes of cereals, milk and dairy products, vegetables, eggs, meat, soybeans and nuts in the two groups all met the minimum requirements of the recommended amount, whereas the intakes of aquatic products, fruits and oils were lower than the minimum requirements of the recommended amount. In terms of iron supplementation, the proportion of iron supplementation in the anaemia patients was higher than that in non-anaemia patients (*p* = 0.020), as shown in Table 5.

**Table 5:** Comparison of daily food intake and iron supplementation status between children with and without anemia.

Item	Anemia(*n* = 31)	No-anemia(*n* = 166)	*P* value	Recommended nutrient intake(g)
Food intake (g/day, Mean ± SD)				
Cereal potatoes	150.58 ± 6.59	156.82 ± 8.29	<0.001	100–150
Milk and dairy products	493.94 ± 12.28	501.10 ± 15.56	<0.001	350–500
Vegetables	104.13 ± 16.82	108.57 ± 9.09	0.034	100–250
Fruits	126.74 ± 15.58	133.34 ± 11.37	0.006	150–250
Eggs	50.61 ± 5.68	54.61 ± 7.17	0.004	50–60
Meat	53.71 ± 14.22	60.33 ± 12.55	0.009	50–75
Oil	18.42 ± 3.12	19.46 ± 5.00	0.263	20–25
Soybeans and nuts	22.74 ± 4.97	18.46 ± 5.35	<0.001	15–25
Aquatic Products	1.03 ± 1.02	2.64 ± 1.26	<0.001	30–50
Iron supplementation (*n*, %)	125	17	0.020	–

Data for food intake are presented as mean ± standard deviation (SD) and were compared using independent sample *t*-tests. Data for iron supplementation are presented as number (*n*) and percentage (%) of children in each group and were compared using the Chi-square test. The recommended nutrient intake is provided as a daily reference range.

### 3.7 Logistic regression analysis

Given the limited sample size (31 anaemia cases), the results of a full multivariate logistic regression model including all significant variables from univariate analysis would be highly unstable and are not presented. Therefore, Table 6 primarily presents the results of univariate logistic regression analyses, which identified several dietary and supplementation factors associated with IDA for preliminary consideration.

**Table 6:** Univariate logistic regression analysis of factors associated with iron deficiency anemia.

Influencing factor	B	S.E.	Wald *χ^2^*	*P* value	*OR*	*OR*(95%CI)
Ethnicity (Ref: Han)						
Kazakh	1.620	1.000	2.624	0.105	5.055	0.712–35.912
Cereal and potatoes (per 1-g/day increase)	−0.080	0.041	3.776	0.052	0.923	0.851–1.001
Milk and dairy products (per 1-g/day increase)	−0.045	0.021	4.521	0.033	0.956	0.918–0.997
Vegetables (per 1-g/day increase)	−0.021	0.025	0.690	0.406	0.980	0.933–1.028
Fruits (per 1-g/day increase)	−0.025	0.023	1.205	0.272	0.975	0.932–1.020
Eggs (per 1-g/day increase)	−0.064	0.047	1.852	0.174	0.938	0.855–1.029
Meat (per 1-g/day increase)	−0.079	0.027	8.494	0.004	0.924	0.877–0.975
Soybeans and nuts (per 1-g/day increase)	0.149	0.060	6.271	0.012	1.161	1.033–1.305
Aquatic products (per 1-g/day increase)	−0.991	0.262	14.292	0.001	0.371	0.222–0.620
Iron supplementation (Ref: No)						
Yes	1.465	0.646	5.143	0.023	4.326	1.220–15.338

OR, odds ratio; CI, confidence interval; Ref, reference category. This table presents the results of univariate analysis. For categorical variables (Ethnicity, Iron supplementation), the OR represents the risk compared to the reference group. For continuous dietary variables, the OR represents the change in risk per 1-gram increase in daily intake.

To provide a more robust and reliable estimate while controlling for potential confounding, a parsimonious multivariate model was constructed, adhering to the rule of thumb of EPV > 10. This limited the model to a maximum of three predictors. The model included the three most clinically significant and statistically robust factors from the univariate analysis: meat intake, aquatic products intake, and iron supplementation status. The results of this adjusted model confirmed that lower meat intake [adjusted odds ratio (aOR) = 0.92, 95% CI: 0.87–0.97], lower aquatic product intake (aOR = 0.35, 95% CI: 0.20–0.59) and failure to take iron supplements (aOR = 5.10, 95% CI: 1.40–18.60) remained independent and significant predictors of IDA.

## 4 Discussion

This cross-sectional study demonstrates a high prevalence of IDA (15.7%) among children in Barkol County, significantly higher than the national average (5). This finding is consistent with the global consensus that IDA remains a major public health issue, particularly in developing regions (10). The burden was disproportionately higher among Kazakh children than Han children, underscoring the role of ethnicity-specific factors within this high-prevalence region.

### 4.1 Key findings and comparison with existing literature

This prevalence rate is consistent with the known elevated risk of anemia in Western China's rural areas and with reports from Xinjiang, where IDA accounts for a high proportion of childhood anemia (6). Our findings add critical nuance by demonstrating a significant ethnic disparity in IDA risk even within this high-prevalence setting. The disproportionately higher burden among Kazakh children compared to their Han counterparts lends further support to meta-analyses linking ethnic minority status to an increased risk of IDA in China (11).

The analysis of dietary factors revealed that lower intakes of meat and aquatic products were significant risk factors for IDA. This is consistent with global evidence that low intake of iron-rich foods, such as meat, is a key risk factor (12) and that vitamin C-rich foods eaten with iron-containing foods can enhance absorption (13). The critical role of animal-sourced foods in preventing IDA is well-established, as they provide highly bioavailable heme iron (14). Conversely—and somewhat unexpectedly—a higher intake of soybeans and nuts was associated with an increased risk of IDA. This may be explained by the high phytate content in these foods, which can inhibit non-heme iron absorption (15), particularly in a diet that may be low in enhancers of iron absorption, such as vitamin C (16). This complex interaction highlights that the overall dietary pattern, rather than single nutrients, determines iron status. The imbalance of dietary structure in children can affect normal body metabolism (17), and dietary fibre can indirectly shape the gut microbiota (18, 19), potentially influencing iron absorption.

### 4.2 Interpretation in the local context and implications

The elevated risk among Kazakh children can be interpreted through the lens of their traditional pastoral lifestyle and diet (6, 20). The characteristic high consumption of milk tea, which contains tannic acid that can precipitate iron salts and hinder iron absorption (20), creates a significant barrier. This is compounded by potentially lower consumption of vitamin C-rich fruits and vegetables and aquatic products, forming a dietary pattern suboptimal for iron bioavailability. This pattern of micronutrient insufficiency, particularly involving vitamin A and E, has been linked to IDA in other studies (16), suggesting a broader nutritional imbalance. This study's finding that aquatic product intake was far below recommended levels across all children aligns with this traditional diet structure.

These findings suggest that interventions must be culturally tailored. Short-term strategies could include targeted iron supplementation programmes, as the failure to supplement iron was a significant risk factor in this study, consistent with previous findings (11). Such interventions are crucial across diverse global contexts, from low- to high-income countries (14). For long-term impact, nutrition education should focus on improving dietary diversification and incorporating practices that enhance iron absorption. This is crucial, as studies have shown that intensive nutritional education can improve iron status in infants (21). Effective nutrition education should be grounded in an understanding of local dietary patterns and nutritional status (15, 22). Improving women's education is also a critical strategy, as a higher level of maternal education is associated with better nutritional practices regarding IDA (23). Furthermore, addressing specific nutritional gaps, such as the provision of adequate whey protein and micronutrients in complementary foods, can form part of a comprehensive strategy (24, 25).

### 4.3 Limitations

This study has several limitations. First, its cross-sectional nature precludes the establishment of causal relationships. The sample was drawn from a specific pastoral and agricultural county in Xinjiang. Therefore, although the results provide important insights into the ethnic disparity and dietary risk factors for IDA in this setting, their generalisability to other regions of China with different socioeconomic and dietary patterns may be limited. Second, the use of retrospective dietary recall may introduce memory bias. Finally, the sample size, though adequate for initial description, limits the complexity of multivariate models that can be reliably fitted. Future research with a larger, prospective cohort design is warranted to confirm these associations and explore causal pathways. Future studies should also consider including measures of inflammation and gastrointestinal health, which are known influencers of iron status (12, 26), and explore the generalisability of these findings in other pastoral populations (27).

In summary, this study identifies a high burden of IDA among children in Barkol County, with Kazakh children being at particularly high risk. The findings highlight the importance of ethnicity-specific dietary patterns and the need for interventions that move beyond simply increasing iron intake to address factors affecting iron bioavailability. Public health efforts should combine short-term nutritional support with long-term strategies aimed at improving dietary diversity and nutrition knowledge within the context of local culture, as paying attention to the prevention and treatment of IDA in children is of paramount importance (28).

## 5 Conclusion

The prevalence of IDA among children aged 3–14 years in Barkol Kazakh Autonomous County was 15.7%, with a significantly higher burden observed in Kazakh children. After adjusting for key confounders in a statistically stable model, the primary modifiable risk factors identified were insufficient intake of meat and aquatic products, and failure to take iron supplements.​ These findings underscore the necessity for targeted public health interventions aimed at improving dietary iron quality and promoting iron supplementation in this population.

## Data Availability

The original contributions presented in the study are included in the article/Supplementary Material, further inquiries can be directed to the corresponding author.
