# Supplementary material for: The investigation and analysis of nutritional iron deficiency anaemia in Kazakh children
Source: Front Pediatr. 2026 Jan 12;13:1654351. doi: 10.3389/fped.2025.1654351 (PMC12832677; doi:10.3389/fped.2025.1654351)
Supplement: Supplementary file 1 [file Supplementaryfile1.docx]

**Supplementary Figure 1.** The flow of participants


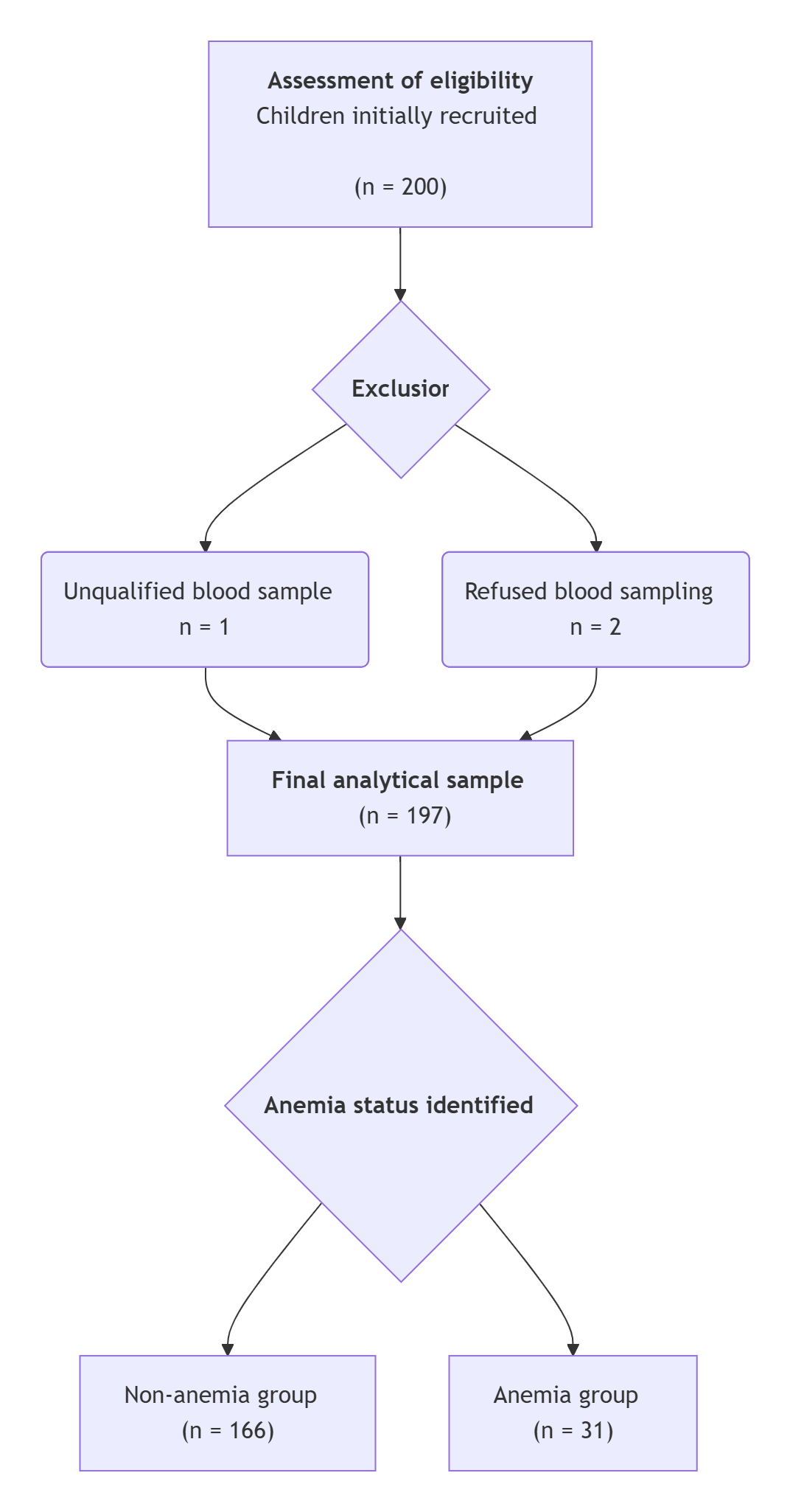


This flowchart illustrates the screening process for study subjects. Ultimately, 197 children were included in the data analysis, of whom 31 were diagnosed with anaemia and 166 were non-anaemic.
